# Side Effect Patterns in a Crossover Trial of Statin, Placebo, and No Treatment

**DOI:** 10.1016/j.jacc.2021.07.022

**Published:** 2021-09-21

**Authors:** James P. Howard, Frances A. Wood, Judith A. Finegold, Alexandra N. Nowbar, David M. Thompson, Ahran D. Arnold, Christopher A. Rajkumar, Susan Connolly, Jaimini Cegla, Chris Stride, Peter Sever, Christine Norton, Simon A.M. Thom, Matthew J. Shun-Shin, Darrel P. Francis

**Affiliations:** a National Heart and Lung Institute, Imperial College London, London, United Kingdom; b Lipid and Cardiovascular Risk Service, Imperial College Healthcare NHS Trust, London, United Kingdom; c Management School, University of Sheffield, Sheffield, United Kingdom; d Division of Care for Long Term Conditions, King’s College London, London, United Kingdom

**Keywords:** crossover trial, drug intolerance, nocebo, side effects, statins, CI, confidence interval, IQR, interquartile range

## Abstract

**Background:**

Most people who begin statins abandon them, most commonly because of side effects.

**Objectives:**

The purpose of this study was to assess daily symptom scores on statin, placebo, and no treatment in participants who had abandoned statins.

**Methods:**

Participants received 12 1-month medication bottles, 4 containing atorvastatin 20 mg, 4 placebo, and 4 empty. We measured daily symptom intensity for each using an app (scale 1-100). We also measured the “nocebo” ratio: the ratio of symptoms induced by taking statin that was also induced by taking placebo.

**Results:**

A total of 60 participants were randomized and 49 completed the 12-month protocol. Mean symptom score was 8.0 (95% CI: 4.7-11.3) in no-tablet months. It was higher in statin months (16.3; 95% CI: 13.0-19.6; *P* < 0.001), but also in placebo months (15.4; 95% CI: 12.1-18.7; *P* < 0.001), with no difference between the 2 *(P =* 0.388). The corresponding nocebo ratio was 0.90. In the individual-patient daily data, neither symptom intensity on starting (OR: 1.02; 95% CI: 0.98-1.06; *P =* 0.28) nor extent of symptom relief on stopping (OR: 1.01; 95% CI: 0.98-1.05; *P =* 0.48) distinguished between statin and placebo. Stopping was no more frequent for statin than placebo *(P =* 0.173), and subsequent symptom relief was similar between statin and placebo. At 6 months after the trial, 30 of 60 (50%) participants were back taking statins.

**Conclusions:**

The majority of symptoms caused by statin tablets were nocebo. Clinicians should not interpret symptom intensity or timing of symptom onset or offset (on starting or stopping statin tablets) as indicating pharmacological causation, because the pattern is identical for placebo. (Self-Assessment Method for Statin Side-effects Or Nocebo [SAMSON]; NCT02668016)

Most people who begin statin therapy abandon it (1, 2, 3), most commonly because of side effects (4,5). More than one-half of the potential benefit of this group of drugs is therefore being lost (6,7). Placebo-controlled trials of over 80,000 participants have found no evidence of an increment in symptoms on statin vs placebo (8, 9, 10). However, when an individual experiences side effects, placebo-controlled information *from others* (even tens of thousands) provides little reassurance.

When consulting a patient experiencing symptoms on statins, physicians must decide whether the statin is responsible. If symptoms disappear and reappear on stopping and restarting, does this prove the statin is the cause?

The SAMSON (Self-Assessment Method for Statin Side-effects Or Nocebo) trial enrolled participants with side effects sufficiently severe to have abandoned statin therapy. Each underwent a protocol of 12 statin, placebo, and no-tablet months. Daily symptom scores were collected using a smartphone app.

In this full report, we show every patient’s full data. We explore the patterns of symptom onset and offset and discuss their diagnostic value.

## Methods

### Study design

This trial used a multiple-crossover, 3-arm, double-blind, placebo-controlled design, recruiting participants from 17 referral centers across the United Kingdom and by self-referral. The study required only 2 study visits, performed at Hammersmith Hospital, London, United Kingdom.

SAMSON was approved by London Brent research ethics committee (REC 15/LO/1761), sponsored by Imperial College London, and registered at ClinicalTrials.gov (NCT02668016) and EudraCT (2015-004109-18).

### Participants

We enrolled participants who had abandoned statins clinically, with no intention of restarting, because of intolerable symptoms of any type arising within 2 weeks of starting. Rechallenging with statins is routine clinical practice when there are symptoms. Many SAMSON participants had tried many times with different drugs or doses, with 12 having tried 4 or more different regimens previously.

For participants, SAMSON was an attractive way to formally document their symptoms beyond doubt, while allowing them to stop as soon as their symptoms became intolerable. Indeed, if this was on the first day of starting tablets, they would only take 4 statin tablets the entire year.

Exclusion criteria (Supplemental Appendix) included concomitant fibrates or dangerous side effects, eg, rhabdomyolysis. To encourage broad participation, SAMSON did not mandate blood tests and involved only 2 visits, with all other contact by telephone or the smartphone app.

Participants were asked to submit a symptom intensity score each day through the app on a visual analogue scale from 0 (no symptoms) to 100 (worst imaginable symptoms). Of course, there was no expectation that participants should persist with a preventative medication that was giving them symptoms anywhere near “worst imaginable.” Participants could stop taking tablets early in any month if symptoms became intolerable, and this was documented on the app. The next month, they would continue the protocol.

When patients stopped tablets because of intolerable side effects, they were asked to continue submitting daily scores to document symptom recovery, but were informed that their results calculation would continue to use the high symptom burden score (see Supplemental Appendix for details). This reduced their discomfort, without the trial underestimating symptoms on tablets.

### Randomization and masking

Statin and placebo medication and bottles were indistinguishable. Prior to trial commencement, an Imperial College Trials Unit statistician created randomized sequences for each participant, comprising 12 1-month medication bottles: 4 containing statin tablets, 4 placebo, and 4 empty. The statistician then randomly ordered these sequences for sequential allocation, which was performed by a computer. All other trial staff were blinded to these allocations.

### Procedures and medications

The statin was atorvastatin 20 mg film-coated (Ranbaxy). The bespoke placebo was manufactured with identical appearance (GSTT Pharmaceuticals).

### Outcomes

Although patients were encouraged to submit a symptom intensity score every day, there were 2 reasons that the analysis may not have access to a full year of scores. First, a participant may not have submitted a score on a certain day. Such missing data were imputed via multiple imputation (11) (see the Supplemental Appendix, which explains this further, and contains a sensitivity analysis where multiple imputation was not used).

Second, because the protocol allowed patients to stop a month’s tablets early if symptoms became intolerable (to allow them relief of symptoms for ethical reasons), there was a danger of underestimating the symptomatic burden from statins if the raw scores were analyzed, ie, with the decline after early tablet stoppage.

Therefore, to reflect the total symptom burden if tablets had not been stopped, we also used multiple imputation (rather than the patient-submitted scores) for the days after stoppage. In contrast, when the focus was the process of symptom offset (including after early tablet stoppage), the analysis used the patient-submitted scores. The Supplemental Appendix contains further information and a graphical explanation for the rationale of this approach.

The primary endpoint requested by our patient advisory group had the “nocebo ratio” defined as follows:

$$\mathrm{Nocebo}\mathrm{ratio}=\frac{\mathrm{Symptom}\mathrm{intensity}\mathrm{on}\mathrm{placebo}-\mathrm{Symptom}\mathrm{intensity}\mathrm{on}\mathrm{no}\mathrm{tablets}}{\mathrm{Symptom}\mathrm{intensity}\mathrm{on}\mathrm{statins}-\mathrm{Symptom}\mathrm{intensity}\mathrm{on}\mathrm{no}\mathrm{tablets}}$$

The original intention was to calculate this ratio individually for each participant. However, some patients had extreme or indeterminable ratios, caused by the denominator of the ratio approaching or equaling zero, making the nocebo ratios very far from normally distributed. An independent statistician therefore recommended pooling the participants before calculating this nocebo ratio.

We therefore now report the marginal means of symptom intensity during statin, placebo, and no-tablet months, which remain valid for statistical analysis and hypothesis testing, and alongside this we report the resulting nocebo ratio.

### Statistical analysis

We modeled change and variation in symptom intensity during and between statin, placebo, and no-tablet months, using mixed (multilevel) linear models. A sequence of 4 models was run. First, an “unconditional” model, which simply separated symptom intensity variance into within-month, between-month, and between-subject components, provided a baseline for subsequent model comparison. Second, we added a fixed effect of treatment to explain variance between months. Third, we allowed the effect of treatment to vary between subjects. Finally, we fine-tuned the model by adding an autoregressive (lag) effect within subjects. The effect size and statistical significance of the treatment effect, and post hoc tests between the marginal means for each treatment type, provided a test of our primary hypothesis, namely whether symptom intensity would differ significantly between the 3 types of months.

As there were no prior data on the relative size of nocebo and statin side effects, we followed the advice of Maas and Hox to have a sample of at least 50 participants to help ensure unbiased estimates of the treatment-level SEs (12).

For a month of data to be included in the numerical analysis, the patient had to have reported at least 10 symptom scores. Participants who had to stop their tablets early because of intolerable symptoms but continued submitting symptom scores were not excluded from the analysis, but their symptom scores following tablet cessation were not used. Within participants who satisfied these inclusion criteria, any further such attritional missing data were handled using multiple imputation by chained equations (used for the primary analysis) (11). The Supplemental Appendix explains the rationale for this approach in greater detail. To ensure the robustness and examine the sensitivity of the results to this choice, we reran the analyses with the missing observations simply excluded.

These statistical analyses and prestudy sample size calculations were performed by an independent statistician (C.S.) using SPSS version 25 (IBM)—this software was also used to manage the data.

To assess whether the severity of symptoms or the extent of relief of symptoms on stopping tablets predicted the identity of a tablet as statin or placebo—or if the rate of stopping tablets was different between statin and placebo—we used a generalized linear mixed binomial (logit) model, fitted using the lme4 package and the R statistical programming language (R Foundation).

## Results

### Participants

**Figure 1:**
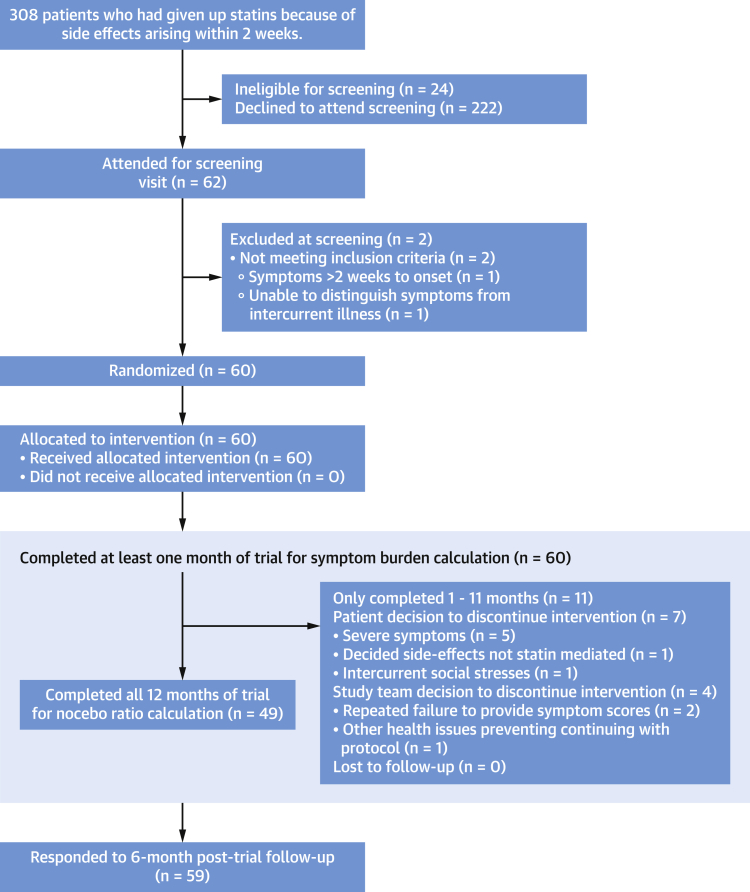
CONSORT Diagram Patient flow through the trial.

A total of 62 participants who appeared eligible attended Hammersmith Hospital for screening between May 1, 2016, and March 1, 2019. Of these, 60 were eligible and randomized (Figure 1).

The mean age of randomized participants was 65.5 ± 8.6 years. All had abandoned statins after trying a median of 2 different statins (IQR: 2 to 3). Median previous statin duration was 1.06 years (IQR: 0.13 to 3.30 years). In 46 (77%) of the randomized participants, the indication for a statin was primary prevention. Full baseline characteristics are shown in Table 1.

**Table 1:** Baseline Participant Characteristics

Age, y	65.5 ± 8.6
Range	37-79
<40	2 (3.3)
40-60	8 (13.3)
>60	50 (83.3)
Sex	
Male	35 (58.3)
Female	25 (41.7)
Ethnicity	
White	54 (90.0)
Black	1 (1.7)
Asian	3 (5.0)
Mixed	2 (3.3)
Height, cm	169 ± 8
Weight, kg	82.0 ± 19.0
BMI, kg/m^2^	29.1 ± 6.7
Number of statins previously tried	2 (2-3)
1	13
2	24
3	11
4	7
5	5
Previous statin duration, y	2.84 ± 4.65 1.06 (0.13-3.30)
Systolic blood pressure, mm Hg	139.1 ± 17.3
Diastolic blood pressure, mm Hg	77.5 ± 8.9
LDL-C, mmol/L	4.16 ± 1.07
Current indication for statin	
Primary prevention	46 (76.7)
Secondary prevention	14 (23.3)
History of diabetes	4 (7.0)
QRISK-2 (18) 10-year risk (primary prevention participants only), %	24.3 ± 13.6
Number of concomitant medications	4.72 ± 3.28

Values are mean ± SD, n (%), n, or median (interquartile range), unless otherwise indicated.

BMI = body mass index; LDL = low-density lipoprotein.

The most common symptoms causing statin abandonment before enrollment were “muscle ache” (n = 36, 60%), “fatigue” or “tiredness” (n = 9, 15%), and “cramps” (n = 6, 10%). All serious adverse events, and nonserious adverse events, judged “severe” or “life-threatening or disabling” are shown in the Supplemental Appendix.

### Symptom scores for statin, placebo, and no treatment

**Figure 2:**
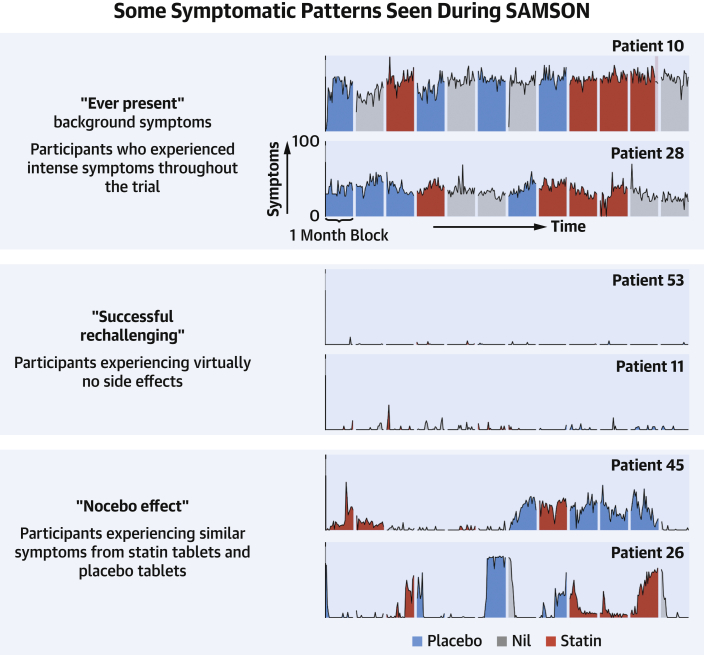
Every Daily Score for 6 Exemplar Patients From the Trial The **vertical axes** represent symptom scores; the **horizontal axes** represent time (days separated into 12 monthly intervals). Symptom intensity bars are colored **gray** in no-tablet months, **blue** in placebo months, and **red** in statin months. **Lighter shaded** regions indicate that patients have stopped tablets early for that month caused by intolerable symptoms. Each participant’s data is labeled by their trial number. Full data for all 60 randomized participants are shown in the Supplemental Appendix.

Of the 60 patients, 49 patients completed the full 12-month protocol. The complete data from 6 exemplar patients are shown in Figure 2, and the data for all patients are in the Supplemental Appendix.

The marginal mean symptom score from the mixed model was 8.0 during the no-tablet months (95% CI: 4.7-11.3), 15.4 during the placebo months (95% CI: 12.1-18.7; *P* < 0.001 vs no-tablet months), and 16.3 during statin months (95% CI: 13.0-19.6; *P* < 0.001 vs no-tablet months; *P =* 0.39 vs placebo months), as shown in the Central Illustration, left panel. Full mixed model results are shown in the Supplemental Appendix.

**Central Illustration:**
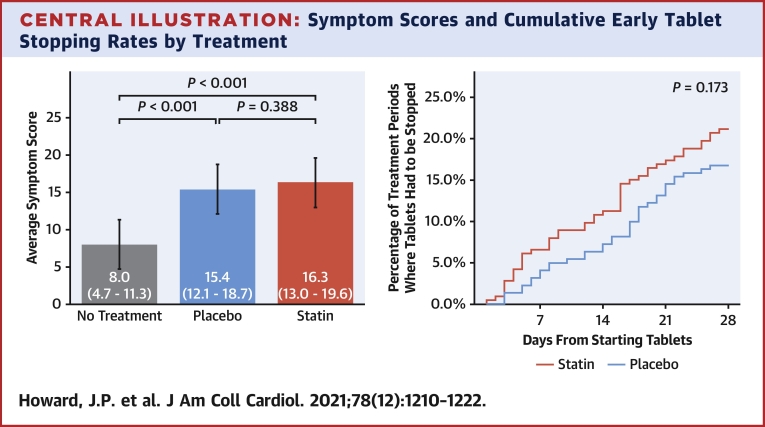
Symptom Scores and Cumulative Early Tablet Stopping Rates by Treatment **(Left)** The mean symptom scores across the 3 treatment types (statin, placebo, and no treatment). **Whiskers** indicate the associated 95% CIs. **(Right)** The cumulative rate of stopping tablets for patients starting a statin **(red)** or placebo **(blue)** after a no-tablet month. *P* value derived from a mixed-effects logistic regression model.

The original primary endpoint calculation agreed with the patient advisory group was of individual nocebo ratios from each participant. As discussed previously (13), the denominator (statin symptoms beyond no tablet) was unexpectedly small or negative in some participants with low symptom burdens, causing the ratios to become extreme and certainly not normally distributed. For full disclosure, calculating the nocebo ratio using individual-patient nocebo ratios yielded 2.2 (95% CI: −62.3 to 66.7).

An independent statistician (C.S.) therefore recommended calculating the ratio from pooled patient data. This nocebo ratio was 0.90.

### Early stopping of tablet months caused by intolerable symptoms

In total, 31 of 60 patients stopped at least 1 tablet month early (comprising 26 stopping statin, and an overlapping 23 stopping placebo). A total of 84 tablet months were stopped early: 46 of 213 statin months (21.6%), with median time to stopping of 15 days (IQR: 6-20 days), and 38 of 221 placebo months (17.2%), with median time to stopping of 18 days (IQR: 9-21 days).

Stopping was not significantly more frequent for statin than placebo (OR: 1.48; 95% CI: 0.85-2.62; *P =* 0.173) (Central Illustration, right panel). There were 8 patients who only stopped a statin month early, and 5 who only stopped a placebo month early.

### Participants who did not complete the trial

In all, 11 participants did not complete the full 12 months of the trial. Five withdrew because of severe symptoms. Participant 17 withdrew after an increasingly symptomatic placebo month. Participant 22 had extreme symptoms immediately in their second and third statin months, but not their first. Participant 40 experienced marked symptoms during their 2 statin months but withdrew from the trial before trying a placebo month. Participant 42 stopped 1 placebo month and 1 statin month early. Finally, participant 54 stopped 2 placebo months early, but not their only statin month.

### Time-course of onset and offset of symptoms

**Figure 3:**
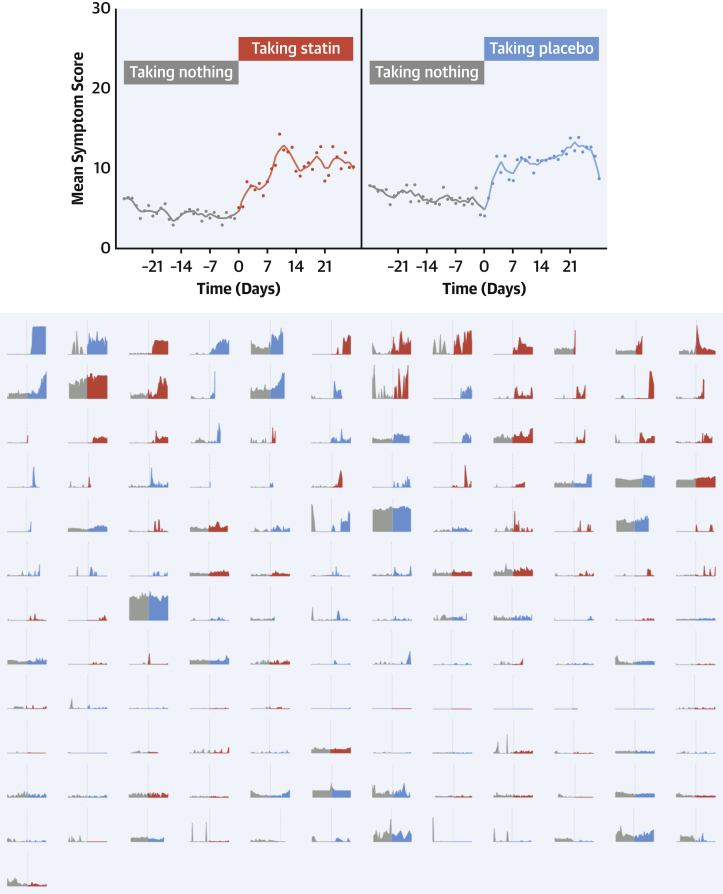
Symptom Time Course in Days Before and After Starting Tablets **(Top)** The symptom pattern averaged across all patients. **(Bottom)** The symptom scores during each unique 2-month period where a patient transitioned between a no-tablet month **(gray)** and a tablet month (**red** for statin, **blue** for placebo). The **dotted vertical line** shows the time of starting tablets. These periods are arranged so the largest increase in symptom scores on starting tablets is in the **top left**, and the lowest in the **bottom right**.

We studied whether the intensity of symptom onset when starting tablets indicated whether that tablet was statin (Figure 3). The top panel of Figure 3 shows the time course of symptoms, averaged across all patients. The lower panel shows full raw data of every 2-month period where a participant transitioned from a no-tablet (gray) to a tablet month (red for statin, blue for placebo), ordered by the increment in intensity.

**Figure 4:**
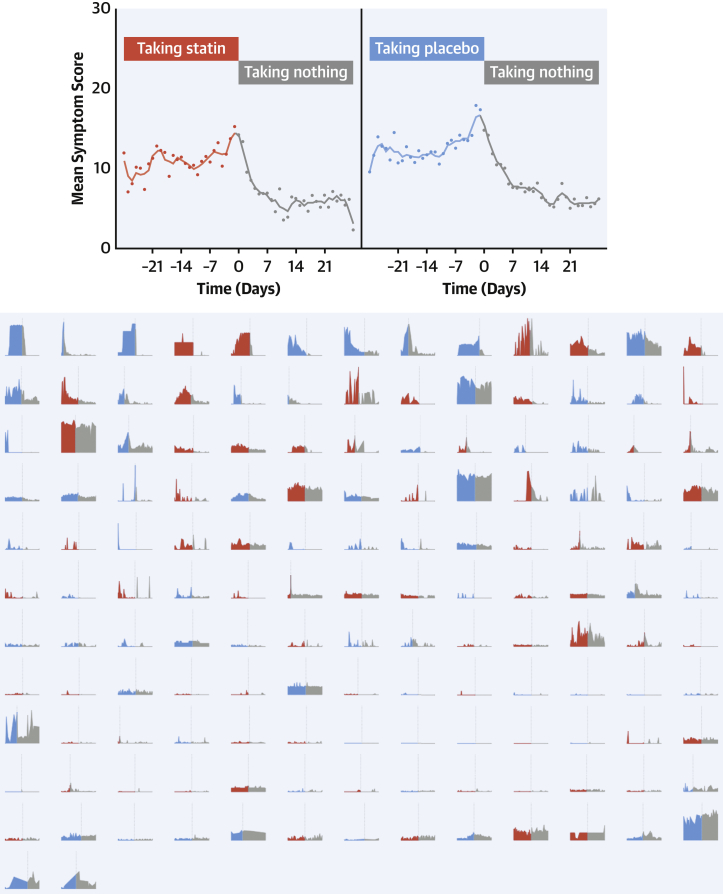
Symptom Time Course in Days Before and After Stopping Tablets **(Top)** The symptom pattern averaged across all patients. **(Bottom)** The symptom scores during each unique 2-month period where a patient transitioned between a tablet month (**red** for statin, **blue** for placebo) and no treatment **(gray)**. The **dotted vertical line** shows the time of stopping tablets. These periods are arranged so the largest increase in symptom scores on starting tablets is in the **top left**, and the lowest in the **bottom right**. Because patients could stop tablets early if they experienced intolerable symptoms, some treatment periods are curtailed.

Intensity of symptom onset did not predict whether the tablet was statin or placebo (OR: 1.02; 95% CI: 0.98-1.06; *P =* 0.28). Figure 4 mirrors this for symptom relief on stopping tablets. Because some tablet months were stopped early, the 2-month periods in the lower panel of Figure 4 sometimes have an earlier transition than scheduled.

The magnitude of symptom relief did not predict whether the tablet was statin or placebo (OR: 1.01; 95% CI: 0.98-1.05; *P =* 0.48). Symptom relief was strikingly rapid. Scores fell by more than one-half within 3 days of stopping tablets on 55% of occasions, regardless of whether the tablet was statin (50% of occasions) or placebo (60% of occasions).

### Restarting clinical statins

At 6 months after each participant’s scheduled completion of the trial, 30 of 60 (50%) had successfully restarted statins. The full baseline characteristics of these patients are shown in the Supplemental Appendix. A further 4 participants said they planned to restart statins, and 1 was uncontactable. Of the remaining 25, reasons given for not restarting statins were as follows: side effects (n = 18), cholesterol spontaneously improved (n = 4), recollection that statins had previously not reduced their cholesterol (n = 1), newly diagnosed neurodegenerative disorder (n = 1), and considering themselves “too old” (n = 1).

Of the 30 participants who successfully restarted statins after receiving their individualized trial results (involving a discussion with a trial doctor and nurse), 10 had stopped tablets during the trial because of intolerable side effects.

## Discussion

Despite having permanently abandoned statin tablets because of intolerable side effects, most participants could nevertheless complete a 12-month multiple-crossover protocol intended to verify these side effects and identify their origins. These side effects predominantly arose from taking a tablet, rather than from the statin within it.

Early cessation of tablets was almost as frequent for placebo as for statin (17.2% vs 21.6%). These results of SAMSON lie in between those from GAUSS-3 (Goal Achievement After Utilizing an Anti-PCSK9 Antibody in Statin Intolerant Subjects-3) (14) (15% vs ∼22% at 28 days) and the recently published StatinWISE (A series of randomized controlled N-of-1 trials in patients who have discontinued or are considering discontinuing statin use due to muscle related symptoms to assess if atorvastatin treatment causes more muscle symptoms than placebo) (15) (19.5% vs 17%). Rates are lower in statin-naive subjects, such as in the STOMP (The Effect of Statins on Skeletal Muscle Function and Performance) trial (16), which had no discontinuation of statin or placebo caused by side effects.

Symptom intensity in SAMSON was also similar between placebo and statin (15.4 vs 16.3 on a visual analogue scale of 1-100), and both were worse than no tablets (8.0). Similarly, in StatinWISE, the results were 1.68 vs 1.85 on a visual analogue scale of 1-10. These trials explain the paradox of no difference in symptomatic side effects between statins and placebo in 80,000 randomized controlled trial participants (8), despite side effects being the commonest reason for statin discontinuation (4,5).

Only SAMSON has no-tablet periods. It can therefore show whether the symptoms experienced when taking a placebo were actively caused by it, rather than being ever-present background symptoms. The nocebo ratio of 90% found in SAMSON does not have a counterpart in the other trials, as they do not have equivalently documented no-tablet periods.

### Were the participants really intolerant of statin tablets?

Superficially, it may appear that the trial has failed to recruit participants with true intolerance. However, it should be remembered that they had self-reported intolerance based on experience solely with statin tablets, ie, no access to blinded matched placebo. The trial confirmed clear (*P <* 0.0001) intolerance of statin tablets: the main surprise was an equally clear intolerance of placebo tablets (*P <* 0.0001), in a population who had been advised during the informed consent process that they would be receiving statin, placebo, and no-tablet periods. The intolerance was severe: 26 of 60 had to stop a statin month early. However, 23 of 60 had to stop a placebo month early. It is clear that this cohort were indeed intolerant of statin tablets, but also that the source of the intolerance was primarily the tablet, not the statin.

### The need for a planned schedule and symptom tracking

Because SAMSON was an exercise in personalized medicine, each participant’s full daily data are shown in the Supplemental Appendix, and they reveal several patterns. Figure 2 shows 6 exemplar patients whose symptom scores we will now cover in more detail. Some participants (eg, participant 53) essentially experienced no side effects during the trial, despite intolerable side effects with statins previously. Others (eg, participant 10) had intense symptoms throughout, regardless of whether they were taking tablets or not.

These 2 patterns represent situations where symptoms not caused by taking statins have been previously attributed to them. In participant 53, these symptoms were presumably an intercurrent phenomenon and have now disappeared. In participant 10, they are “ever-present” and not exacerbated by statins.

For a patient to discover this for themselves requires a formal schedule with systematic symptom tracking, otherwise they might stop tablets only when they have bad symptoms (and can only get better), and restart them only when they feel well (and can only get worse). This inevitably leads them to believe that stopping the tablets alleviated the symptoms, and restarting exacerbated them.

### The need for a placebo

The third pattern was statin and placebo inducing similar symptoms (eg, participant 45). These symptoms are genuine, but are caused by the nocebo effect, rather than the statin. Patients and clinicians do not have access to matched placebo in clinical practice. Unfortunately, therefore, even with a careful protocol and meticulous documentation, they will misattribute this nocebo effect as evidence of statin intolerance.

### The need for no-tablet periods

The classical pattern of a crossover trial is to alternate between active and placebo medications. However, to test side effects, this is unwise. If SAMSON had only active and placebo months, this would have caused 2 problems.

First, in patients with symptoms during both statin and placebo months, we would not be able to discriminate between the nocebo effect and ever-present background symptoms. Second, a theoretical concern might be that SAMSON did not wait long enough for the statin side effects to wash out, and these were causing the high placebo scores. The inclusion of no treatment periods mitigates this concern because the no treatment months had significantly fewer symptoms (*P <* 0.001) than the placebo months.

### The etiology of statin side effects

One possible origin of statin side effects is a direct pharmacological effect. Statins are intended to interfere with liver metabolism, reducing cholesterol production. Placebo-controlled trials show that statins certainly elevate blood levels of liver enzymes, but do not prompt symptoms, until the participant is unblinded and discovers they are taking a statin (9). In SAMSON, however, side effects were not greater on statin than placebo.

A second possibility is that patients starting statins may notice a chance increment in fluctuating background symptoms, and correctly remark that they have increased. SAMSON excluded this by having no-tablet periods and multiple periods of statin and placebo dosing.

A third possibility is unintentional creation of a false association through patients or doctors trying to test causation by starting and stopping tablets as an informal experiment. Unfortunately, without a preplanned schedule, the statin tends to be stopped when symptoms are maximal (and naturally tend to decline) and restarted when symptoms have resolved (and can only get worse). These informal experiments replace uncertainty with confident, incorrect conclusions. SAMSON’s prearranged schedule of monthly treatment periods in a random order eliminates this error.

Finally, patients may be primed to expect symptoms. Sources of priming include reports from friends and family, media and internet coverage (17), and side effects listed in leaflets (that conventionally do not compare active with placebo) (18). A clinician responding to symptom reports by changing the dosage, frequency, or agent reinforces a patient’s belief that the statin was the cause of the symptom. SAMSON tested for this by having both no-tablet and placebo tablet arms, which reveals the expectation of side effects (ie, nocebo) to be the dominant contributor, because symptoms were much worse on placebo tablets than no tablets (*P <* 0.001), and no different between placebo and statin *(P =* 0.388). Furthermore, 10 participants who had to stop tablet months early managed, after discussion of their personalized results, to successfully restart statins.

### Danger of current practice of informal experiments

Prompt onset and offset of symptoms after starting and stopping tablets is often interpreted by patients and clinicians as evidence of causation. Our data indicate that this is true, but because the kinetics are identical between placebo tablets and statin tablets (Figures 3 and 4), the causation is from taking a tablet, rather than from the tablet being a statin.

The danger of the informal experimentation that is currently recommended in North America (19), Europe (20), and the United Kingdom (21) is that even if there is a preplanned schedule and daily symptom scoring, without a placebo it is inevitable that nocebo symptoms will be misattributed to the statin. This phenomenon would unfortunately contribute to the 50% of statin starters who stop.

Just as unfortunate current clinical practice might entrain a nocebo response, experience of multiple crossovers with no-tablet periods might be helpful to reverse the process, desensitizing patients to this nocebo effect.

### Study implications

The 3-arm multiple-crossover design (including no-tablet and placebo periods) allows individualized verification of the existence of side effects of statin tablets, and exploration of the contributions of taking a tablet vs the tablet being a statin. The results have important implications for patients and physicians when symptoms are experienced on statin tablets in routine clinical practice.

The first practical implication is that even severe, convincing, and intolerable symptoms in clinical practice sometimes do not reappear on formal evaluation with daily documentation. This occurred in participants 11 and 53, who had each previously abandoned 2 different statin regimens (Figure 2).

Second, formal documentation of symptom scores sometimes reveals the culprit to be background fluctuations in symptom intensity, regardless of tablets (eg, participants 10 and 60) (Figure 2).

Third, there are verifiable side effects of statin tablets. However, these were similar in intensity between statin and placebo, which means that even reproducible induction of symptoms by statin tablets provides no information about whether the cause is the tablet or the statin component.

Finally, it is wrong to interpret rapid symptom decline after stopping tablets as evidence that the statin was the cause, because the decline is similarly rapid and profound for both statin and placebo.

### Study limitations

The principal limitation of SAMSON is that it only recruited participants with symptoms that arose within 2 weeks of commencing statins. This allowed a trial duration that would be acceptable to most potential participants. To allow many periods of statin, placebo, and no tablets, we made each period 1 month long. To give enough time for symptoms to manifest, we therefore only enrolled participants whose symptoms characteristically arose within 2 weeks. SAMSON only tested a single statin at a single dose. We chose atorvastatin 20 mg daily because this is an inexpensive off-patent agent with satisfactory efficacy data, and is guideline-recommended for primary prevention (22). Finally, SAMSON did not require blood samples to avoid discouraging participation, to prevent delays to stopping tablets, and to maximize clinical applicability.

Future research should address why statins show such a high nocebo effect. One possibility is the dual misfortune of statins commonly being started for primary prevention where there are no symptoms to improve in an age group wherein ill-defined discomfort becomes increasingly common. Florid reports by mass media, unfiltered by scientific discipline, may lead to a vicious cycle where increased vigilance causes increased prevalence and vice versa. Induction of physical symptoms through information is recognized in medicine (10,23). The fact that atorvastatin is now the most prescribed medication (24) has led to the following: 1) there are more patients to make an initial report of symptoms; 2) the media have a greater interest in publicizing them; and 3) individuals have a higher probability of being on the drug and having their attention drawn to symptoms. This triple combination might explain the dramatically higher public perception of side effects with statin tablets than with other medications.

### Flawed original calculation method for primary endpoint

When we designed SAMSON with our patient advisory group, it was felt that it would be easy for participants to understand an expression of the nocebo effect as a proportion of that participant’s overall symptoms from taking statin tablets. We and the patient advisory group assumed that this individual-participant “nocebo ratio” would lie in the range 0%-100%. However, we wrongly assumed that every participant (who had permanently abandoned statin tablets because of side effects) would have substantially higher symptoms on statin tablets than on no tablets, whether that be because of pharmacological reasons or nocebo. Unfortunately, once participants began to exit the trial, it became apparent that some had *lower* symptoms taking statins than not taking statins. This caused the nocebo ratio to have an extraordinarily wide distribution, and made the prespecified calculation highly uninformative. We sought independent statistical advice on the topic and were advised that if we wanted to report the nocebo ratio, we should change the order of steps: pool the participant scores and then calculate the nocebo ratio. This prevented the few patients with unexpected patterns from rendering the measure uninterpretable. It is this revised calculation that we now report: 0.90. However, in full disclosure, we have also reported the result using the original method, with its profoundly wide CIs (2.2; 95% CI: −62.3 to 66.7). Individualized proportional change is not a good formal statistical endpoint for a trial (25), even though patients can understand it.

## Conclusions

Side effects from taking statin tablets are verifiable but are driven by the act of taking tablets rather than whether the tablets contain a statin. The cues and informal experiments patients and clinicians use to test causation can paradoxically confirm a nonexistent association. This error is prevented by a scheduled, 3-armed, crossover trial containing no-tablet periods. Participating in such a protocol allows one-half of patients with reported side effects to successfully restart statins.

Perspectives **COMPETENCY IN PATIENT CARE AND PROCEDURAL OUTCOMES:** In a crossover trial that included no-treatment periods, patients who had stopped statin medication because of side effects exhibited similar frequencies and intensities of side-effect symptoms when starting and similar relief of symptoms when stopping statin and placebo.

**TRANSLATIONAL OUTLOOK:** In future trials, a multiple-crossover trial design that includes a no-treatment arm as well as an active drug and placebo could prove useful in isolating side-effect profiles related to nonpharmacological factors.

## Funding Support and Author Disclosures

This study was funded by the British Heart Foundation (PG/15/7/31235), which had no role in study design, data collection, data analysis, data interpretation, or writing of the report. This study was supported by the National Institute for Health Research Imperial Biomedical Research Centre (BRC) and the Imperial Clinical Trials Unit. The views expressed are those of the author and not necessarily those of the National Institute for Health Research or the Department of Health and Social Care. Dr Howard is supported by the Wellcome Trust, grant number 212183/Z/18/Z. Dr Nowbar is supported by the National Institute for Health Research Academy. Dr Rajkumar is supported by the Medical Research Council, grant number MR/S021108/1. All other authors have reported that they have no relationships relevant to the contents of this paper to disclose.
