# Supplementary material for: Side Effect Patterns in a Crossover Trial of Statin, Placebo, and No Treatment
Source: J Am Coll Cardiol. 2021 Sep 21;78(12):1210–22. doi: 10.1016/j.jacc.2021.07.022 (PMC8453640; doi:10.1016/j.jacc.2021.07.022)
Supplement: Supplemental Data [file mmc1.docx]

Supplementary Appendix

[Inclusion and exclusion criteria 2](#_Toc74122323)

[Rationale for Imputation 3](#_Toc74122324)

[Baseline characteristics of the participants who did not complete the trial 5](#_Toc74122325)

[Comparison of the four multi-level models used in the primary analysis 6](#_Toc74122326)

[Complete data for participants who completed the trial 7](#_Toc74122327)

[Complete symptom data for the participants who did not complete the trial 9](#_Toc74122328)

[Serious adverse events and non-serious adverse events graded ‘severe’ or ‘life threatening or disabling’ 10](#_Toc74122329)

# Inclusion and exclusion criteria

## Inclusion Criteria

- Aged 18 years or older
- Previously taken one or more statins
- Withdrawn from statins because of perceived side effects
- Developed side effects within 2 weeks of initiation
- Clinical indication for statins for primary or secondary prevention of cardiovascular disease or dyslipidemia, taking into account available guidelines criteria but ultimately based on clinician judgment

## Exclusion criteria

- History of any condition that causes chronic pain
- History of severe mental illness (as their experience of symptoms may already be altered)
- Currently taking fibrates (because of the risk of interaction with statins but will not exclude participants taking ezetimibe).
- History of statin intolerance with creatine kinase elevation greater than 5 times the upper limit of normal (ULN)
- History of statin intolerance with anaphylaxis
- History of statin intolerance with myalgia and rise in serum creatine kinase
- History of statin intolerance with rhabdomyolysis
- History of statin intolerance with liver function abnormalities, defined as aspartate aminotransferase (AST) or alanine aminotransferase (ALT) >3 times the ULN
- Currently taking antiretrovirals with known interaction to statins
- Currently taking any drug other than antiretrovirals with known interaction to statins
- Pregnant or breast feeding
- Side effects taking longer than 2 weeks to present (because in such participants much longer blocks of treatment would be required, if the present study is positive such studies will be planned for the future)
- In clinical judgement of study doctor, participant should not be enrolled on the study

# Rationale for Imputation

When designing SAMSON, we did not want potential participants to be discouraged by the thought of severe symptoms for 4 months. We therefore reassured them that they could stop taking tablets during any month if the symptoms became intolerable, even if they had only managed a few days of taking tablets. Even though the symptoms were expected to resolve after stopping tablets, we explained the statistical methods would prevent this from underestimating the symptoms caused by tablets.

The image below explains how multiple imputation can allow patients to stop tablets early, without underestimating the symptom burden caused by the tablets.

| 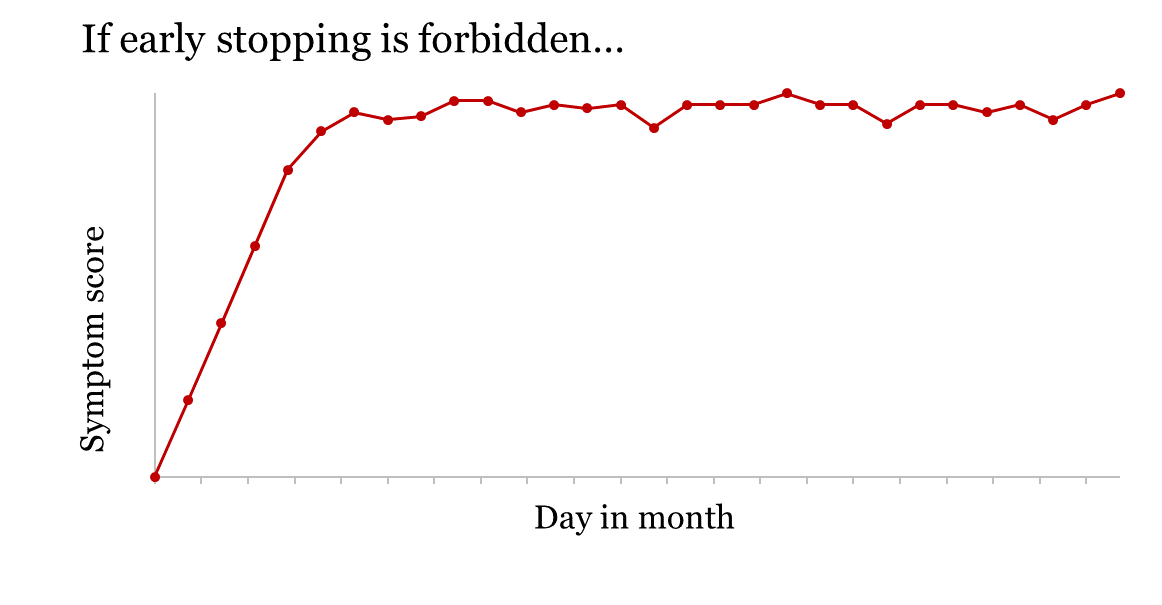 | …the patient reported values would directly reflect symptoms on tablets, but it would be cruel and unrealistic to expect participants to endure this. |
| --- | --- |
| 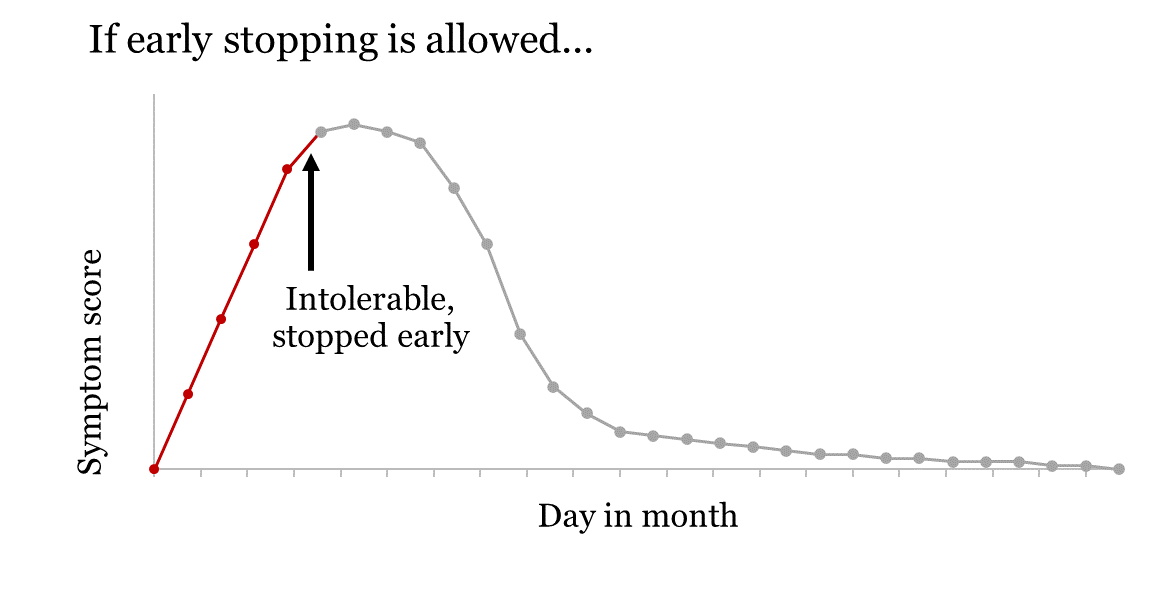 | …participants can get prompt relief from intolerable symptoms, but the data from the latter part of the month will severely underestimate the true burden of side effects from tablets. |
| 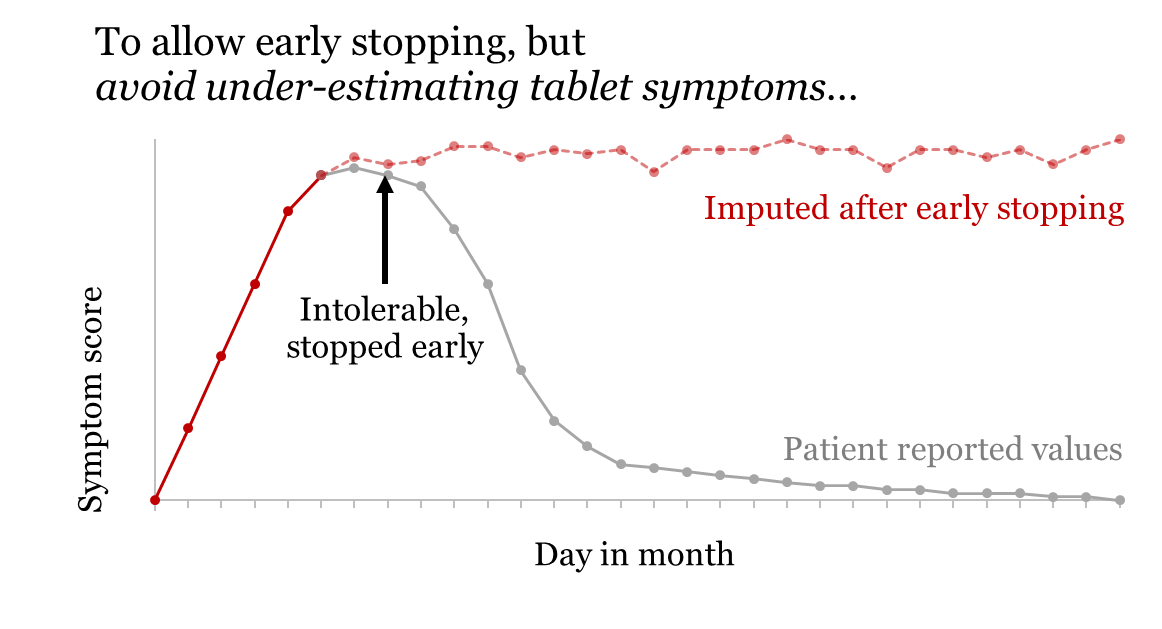 | …we use multiple imputation when calculating the symptom burden from a complete month of tablets, and yet use the directly patient-reported values when calculating the recovery from cessation of tablets. |

## Imputation for days between patient-submitted scores

Patients were encouraged to submit a symptom intensity score every day. For days where a participant did not submit a score, the missing data were imputed via multiple imputation. This was used, on average, 5.8 days per month, per patient.

As a secondary analysis, however, we re-analyzed our data **without** using multiple imputation (with the missing data simply excluded): this results in very similar findings with unchanged conclusions to those reported using multiple imputation. Specifically, this shows a mean symptom intensity was 6.2 during no-tablet months (95% confidence interval 2.7 to 9.7), 11.2 during placebo months (7.7 to 14.7; p<0.0005 versus no-tablet months) and 11.8 during statin months (8.3 to 15.3; p<0.0005 versus no-tablet months; p=0.450 versus placebo months). The corresponding nocebo ratio would be 0.80.

## Imputation for days after early cessation of tablets

Because the protocol allowed patients to stop a month’s tablets early if symptoms became intolerable, there were two analytical methods for scores after an early stoppage. For measurement of average symptomatic burden of statin versus placebo versus no tablet, we used multiple imputation (rather than the patient-submitted scores) for the days after stoppage. In contrast, analysis of symptom offset (relief from stopping tablets) used the patient-submitted scores.

These are two quite different settings. In the former, it is because data is “missing”. Multiple imputation was used to impute, on average, 5.8 days per month per patient.

In the latter, it is because the data is available, but should not be used. This corresponds to, on average, 2.0 days per month per patient.

## Imputation methodology

Multiple imputation was performed using the multiple imputation with chained equations (MICE) method. This was performed using the MICE package in R.[1] This method, also termed “sequential regression multiple imputation” [2] is based around creating a regression model where the dependent variable is the variable of interest (e.g. daily symptom score on a specific day), with associated independent variables (day of month, study month, treatment, and the “raw” symptom scores for other days). This model is then used to predict (impute) the missing data on the variable of interest. These steps are then repeated for the next variable with missing values (e.g. daily symptom score on the next day) and so on until all variables with missing values have been imputed. The original missing values on the first variable are then re-estimated and the process cycles through the list of variables with missing data again. This continues for a chosen number of cycles until estimates for missing values have converged to stability.

[1] Zhang Z. Multiple imputation with multivariate imputation by chained equation (MICE) package. Annals of translational medicine. 2016 Jan;4(2).

[2] Azur MJ, Stuart EA, Frangakis C, Leaf PJ. Multiple imputation by chained equations: what is it and how does it work? International journal of methods in psychiatric research. 2011 Mar;20(1):40-9.

# Baseline characteristics of the participants who restarted statin therapy after the trial

| Age (years) | 64.9(8.23) |
| --- | --- |
| Gender  Male  Female | 22(64%)  8(36%) |
| Ethnicity  White  Black  Asian  Mixed | 28(93%)  0(0%)  1(3%)  1(3%) |
| Height (cm) | 169 (8.46) |
| Weight (kg) | 83.3 (18.81) |
| BMI | 29.1 (6.7) |
| Number of statins previous tried  1  2  3  4  5 | 2 (1 to 3)*  8  14  4  3  1 |
| Previous statin duration (years) | 3.22 (5.39) |
| Systolic blood pressure (mmHg) | 139(13.94) |
| Diastolic blood pressure (mmHg) | 78(13.94) |
| LDL-C (mmol/L) | 3.81(0.93) |
| Current indication for statin  Primary prevention  Secondary prevention | 21 (70%)  9 (30%) |
| History of diabetes | 2 (6%) |
| QRISK-2 (Hippisley-Cox et al. 2008) 10-year risk (primary prevention participants only) | 27% (14.57) |
| Number of concomitant medications | 4.8 (3.53) |

# Comparison of the four multi-level models used in the primary analysis

| Model | Dev* | ΔDev | p | Within month variance | Between month within subject variance | Between subject variance (intercept) | Between subject variance (slope) | Correlation between adjacent days within month |
| --- | --- | --- | --- | --- | --- | --- | --- | --- |
| Unconditional | 143258 | --- | --- | 111.4 | 119.9 | 132.2 | --- | --- |
| Fixed effect of treatment | 143171 | 87.1 | < 0.001 | 111.4 | 103.3 | 134.1 | --- | --- |
| Add random effect of treatment | 143167 | 4.6 | 0.032 | 111.4 | 96.4 | 131.6 | 9.5 | --- |
| Adjust for autoregressive effect within study months (AR1 type autocorrelation) | 132980 | 10189 | < 0.001 | 137.6 | 64.7 | 130.8 | 9.0 | 0.714 |

The deviances of the four multilevel modules assessed.

* Deviance was calculated as the -2Loglikelihood. P values were interpreted using a Chi-square distribution.

# Complete data for participants who completed the trial
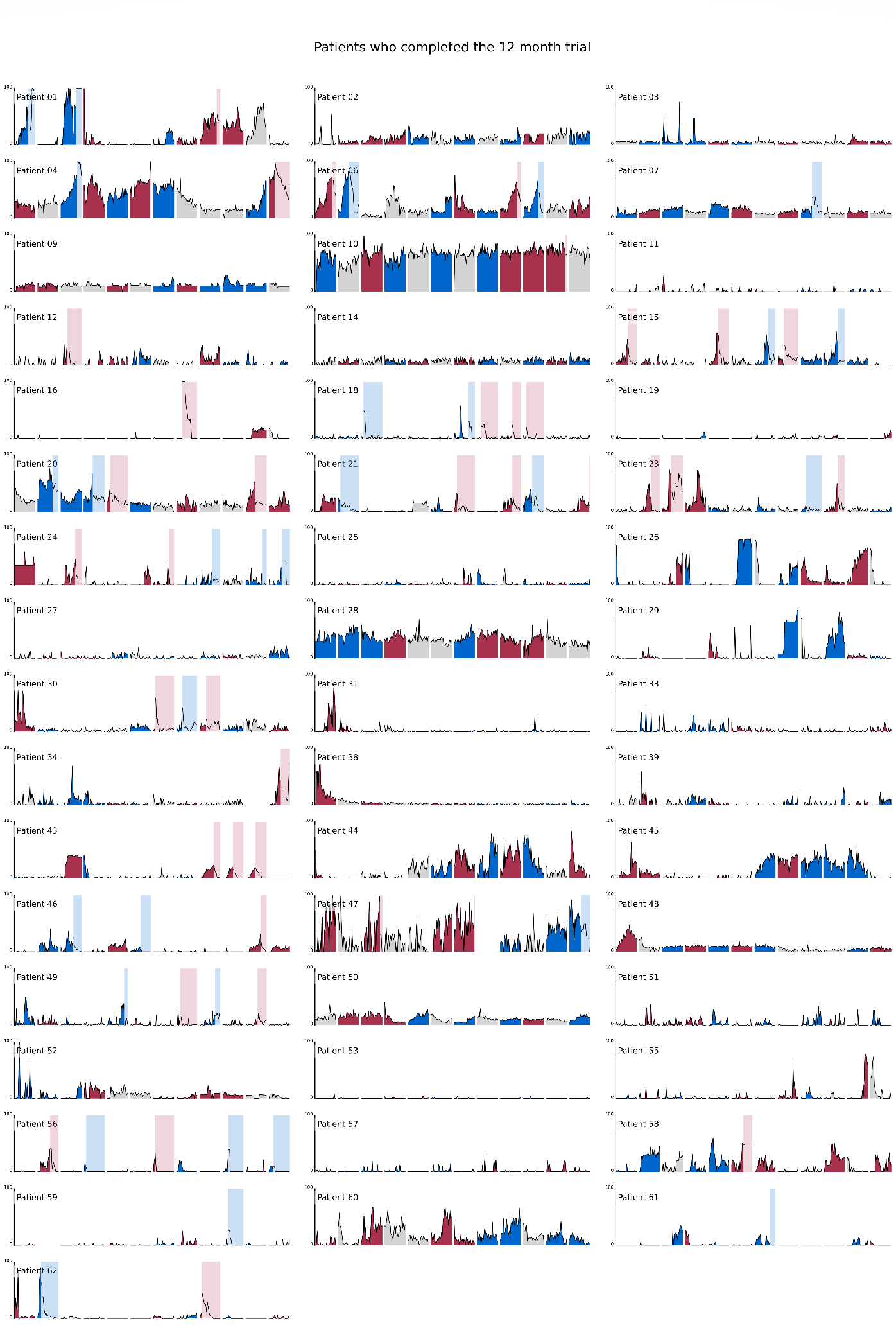


# Complete symptom data for the participants who did not complete the trial


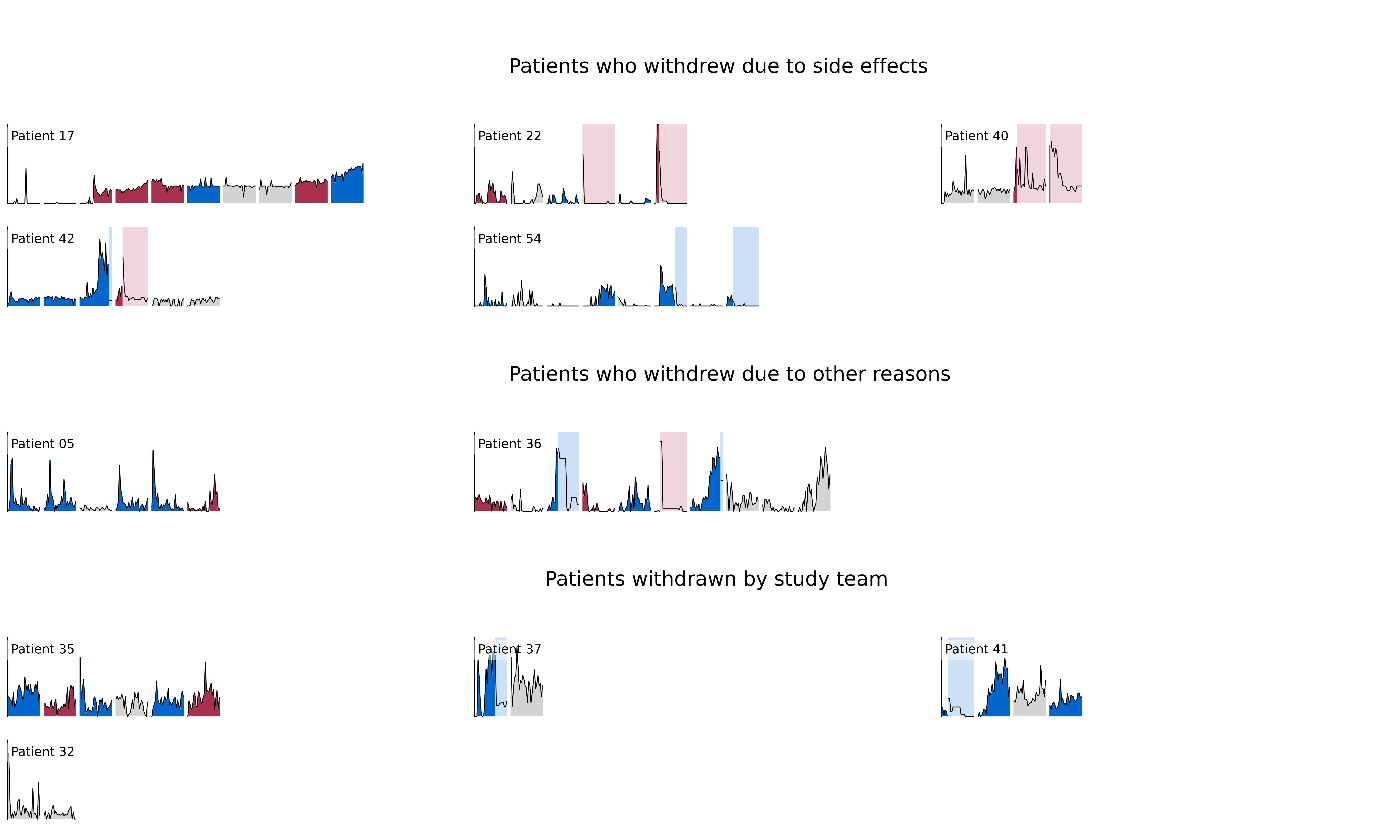


# Serious adverse events and non-serious adverse events graded ‘severe’ or ‘life threatening or disabling’

## Serious Adverse Events

| System organ Class  Preferred Term | Frequency during main study and  6-month follow-up |
| --- | --- |
| Blood and lymphatic system disorders |  |
| Polycythemia | 1 |
| Gastrointestinal Disorders |  |
| Obstructive pancreatitis * | 1 |
| Infection and infestations |  |
| Urinary tract infection | 1 |
| Influenza | 2 |
| Surgical and Medical Procedures |  |
| Tonsillectomy | 1 |
| Cardiopulmonary bypass | 1 |
| Shoulder arthroplasty | 3 |
| Transurethral prostatectomy | 1 |
| Vascular disorders |  |
| Transient Ischemic Attack | 1 |
| Myocardial Infarction | 1 |

## Non-serious Adverse Events, judged ‘severe’ or ‘life-threatening or disabling’

| System organ Class  Preferred Term | Frequency during main study and 6-month follow-up |
| --- | --- |
| General disorders and administration site conditions |  |
| Pain ** | 1 |
| Nervous system disorders |  |
| Multiple System Atrophy | 1 |
| Immune system disorders |  |
| Anaphylactoid reaction *** | 1 |

* Biliary Pancreatitis

** Worsening of pre-existing condition resulting in withdrawal from trial

*** Ferrous iron infusion
